# Unlocking new frontiers in vector control strategies using *Aedes aegypti* microbiota

**DOI:** 10.1186/s13071-026-07304-5

**Published:** 2026-05-05

**Authors:** Houeffa Dokpomiwa, Etienne Bilgo, Anna-Bella Failloux

**Affiliations:** 1 Institut de Recherche en Sciences de La Santé (IRSS), Direction Régionale de l’Ouest, Bobo Dioulasso, Burkina Faso; 2 Institut National de Santé Publique (INSP)/Centre Muraz, Bobo Dioulasso, Burkina Faso; 3 Ecole Doctorale Sciences Naturelles et Agronomie (ED/SNA), Université Nazi Boni, Bobo Dioulasso, Burkina Faso; 4 Institut Pasteur, Université Paris Cité, Arbovirus et Insectes Vecteurs, Paris, France

**Keywords:** *Aedes aegypti*, Microbiota, Vector competence, Mosquito-borne disease, Vector control, Africa

## Abstract

**Background:**

Controlling *Aedes aegypti*, the key vector involved in the transmission of numerous arboviruses, is a major concern, particularly in Africa, where transmission is increasing overall punctuated by annual fluctuations. Traditionally focused on reducing their populations or eliminating their suitable habitats, innovative strategies such as those exploiting microbiota to reinforce existing tools are needed. The microbiota of *Ae. aegypti*, which is composed of diverse symbiotic microorganisms, is involved in their physiology, reproduction, and ability to transmit pathogens, indicating considerable potential for vector control.

**Methods:**

Here, we seek to review the current knowledge on the microbiota of *Ae. aegypti* and its relevance in vector control, with a particular focus on African populations of *Ae. aegypti*.

**Results:**

First, we provide an overview of two major *Aedes* vectors and *Aedes*-borne virus distribution in Africa, their microbiota structure, and some factors likely to influence it, showing that ambient environment is one of the determining factors. Second, we have outlined studies that have explored microbial components involved in the enhancement and attenuation of the vectorial competence of *Ae. aegypti* worldwide, followed by an overview on African *Aedes* mosquito populations. We then examined the impact of global changes on the vector‒microbiota complex, and by extension, on the epidemiology of vector-borne diseases in Africa. Finally, we analyzed the added value of strategies exploiting the mosquito microbiota and the obstacles limiting their large-scale implementation.

**Conclusions:**

Overall, this review highlights the promising use of microbiota for the control of *Ae. aegypti* while identifying future research directions for its large-scale deployment in Africa.

**Graphical Abstract:**

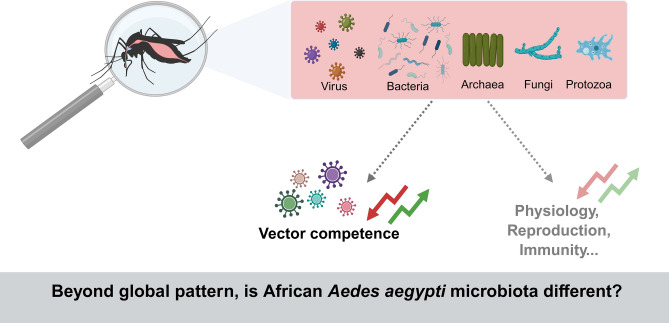

**Supplementary Information:**

The online version contains supplementary material available at 10.1186/s13071-026-07304-5.

## Background

Mosquitoes belong to the family *Culicidae* and comprise three main genera: *Aedes, Anopheles*, and *Culex*. They are involved in the transmission of a wide range of pathogens, including viruses. These viruses are grouped under the nontaxonomic term “arbovirus” (**ar**thropod-**bo**rne **virus**), which is able to replicate in both vertebrate hosts and arthropod vectors. *Aedes* mosquitoes transmit the four most important arboviruses for human health: Zika virus (ZIKV), chikungunya virus (CHIKV), yellow fever virus (YFV), and dengue virus (DENV) [1]. Unlike yellow fever, for which an effective and widely distributed vaccine has long been available, the situation is different for other *Aedes*-borne arboviruses, such as DENV and CHIKV [2]. Although vaccines against these diseases have recently been authorized in some countries, their widespread distribution is still limited by several safety constraints [2–4]. In addition, many candidate vaccines against ZIKV are currently under clinical evaluation; however, none have yet obtained approval by health authorities [5]. Pending scientific advances to strengthen the biological prevention of these arboviruses, prevention efforts rely mainly on vector control measures. These measures aimed to reduce the mosquito population and thus limit virus transmission. However, designing appropriate vector control strategies requires in-depth knowledge of the vector to identify flaws that could be used. Vector control actions can be roughly grouped into three categories: limiting human‒mosquito contact, reducing vector density, and blocking pathogen transmission in mosquitoes [6, 7].

The mosquito life cycle consists of four successive stages during which the mosquito undergoes molting and metamorphosis. From egg to nymph, the mosquito develops in an aquatic environment. The typology and physicochemical properties of larval breeding sites are determining factors in the choice of female oviposition site [8, 9]. In addition, these aquatic habitats contain microorganisms that may affect mosquito physiology and the composition of mosquito microbiota [10, 11]. The microbiota corresponds to a community of symbiotic or pathogenic microorganisms colonizing mosquito organs [12]. It includes bacteria, viruses, fungi, and eukaryotes and is found mainly in the midgut, cuticles, salivary glands, and reproductive organs [13, 14]. It may influence certain factors of mosquito physiology and the ability to transmit pathogens.

**Fig. 1:**
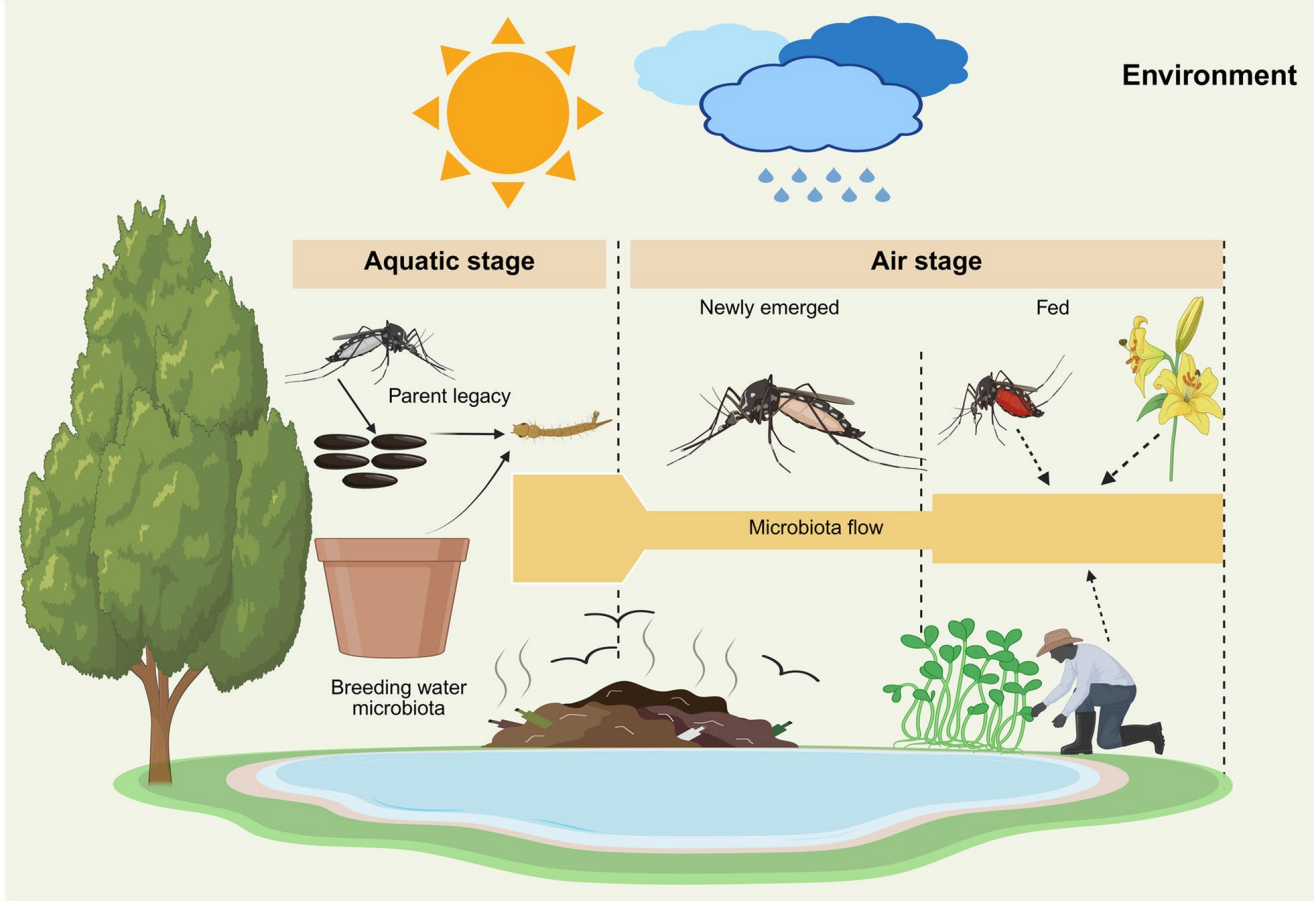
Coevolution of microbial communities with mosquitoes. Created in BioRender. DOKPOMIWA, H. (2025) https://BioRender.com/cy5nskp

During mosquito development, the larval habitat determines the microbiota composition, which changes significantly from larvae to adults [15]. Adult midgut harbors a different composition than the larval water environment [16]. Initially aquatic larvae share breeding water microbial flora but but undergo microbiota shifts during molt [17, 18], retaining only a small fraction at emergence (Fig. 1). Then, adults reshape their intestinal microbial community through sugar and blood meals [14]. Thus, the breeding water and larval microbiota are correlated, while the adult microbiota differs significantly.

Nevertheless, it seems that for each mosquito species, there is a set of microbial taxa referred to as “species-specific microbiota” or “core microbiota,” which refers to a characteristic microbial profile that persist with the species despite environmental variations [19, 20]. This core microbiota could therefore consist of core bacteriome, core virome, etc. Although there is still debate about the existence and definition of core microbiota, as well as their mandatory nature and function, in this study we consider the terms “species-specific microbiota” and “core microbiota” to be interchangeable as long as there are associated with a specie. Thus, the core microbiota defined for a specific species of Culicidae is not necessarily the same as that of all Culicidae in general. Similarly, the core microbiota of a species found in Africa should be similar to those of the same species found outside the continent.

Upon emergence, the mosquito adult, which already hosts a microbiota with some bacteria acquired from the larval habitat, receives multiple sugar meals and blood meals (exclusively females), which are likely to modify the microbiota composition [11, 19]. This dynamic interaction between the mosquito and its environment highlights the importance of understanding how external factors influence its microbiota.

The ambient environment is also a bacterial reservoir favoring the selection and dissemination of bacteria, including those that have developed resistance to antibiotics [21, 22]. Given that mosquitoes, particularly *Ae. aegypti* mosquitoes, live closely with humans, it is important to consider the possible transfer of multiresistant bacteria from the aquatic environment to the microbial flora of mosquitoes. This transfer may affect the life traits of mosquitoes and their capacity to transmit pathogens, particularly through the biological cost of carrying multidrug-resistant bacteria (change in bacteria competitiveness [23]) or through possible changes in the essential functions of the microbiota, although the precise mechanisms and their actual biological relevance remain to be demonstrated.

This review provides a critical discussion on the cohabitation of species-specific core bacteriota with a transient opportunistic microbiota that is highly dependent on the environment and their impacts on vector competence (VC). This overview highlights how these interactions influence vector-borne disease transmission, paving the way for innovative control strategies involving the microbiota. We will focus on African populations of *Ae. aegypti*, as the region is experiencing active transmission of *Aedes*-borne arbovirus, while data on microbiota composition in these native populations remains limited. Synthesizing available knowledge for this topic in this geographical area is therefore essential to identify gaps and guide further research for region-specific vector control strategies.

## Current state

### Overview of *Aedes*, *Aedes*-borne virus distribution in Africa and microbiota structure

Africa represents a particular context in the worldwide epidemiology of *Aedes* mosquitoes, as it is the ancient origin of *Ae. aegypti* [1]. Present in all five regions of the continent, *Ae. aegypti* has two (02) subspecies, *Ae. aegypti aegypti* (*Aaa*) and *Ae. aegypti formosus* (*Aaf*), which differ in their morphology, ecology, and genetics [24]. The *Aaf* subspecies, described as darker and characterized by the absence of white scales on the first abdominal tergite, is known as sylvatic and zoophilic and reproduces in natural habitats such as tree cavities. *Aaa* subspecies is found in urban areas, have at least one white scale on the first abdominal tergite, are anthropophilic, and reproduce in artificial breeding sites [24, 25].

Another major *Aedes* species involved in the transmission of the four *Aedes*-borne viruses is *Aedes albopictus*, an invasive species native to Asia that has recently invaded Africa, leading to its coexistence with *Ae. aegypti* in some countries [26, 27]. It exploits a wide range of larval habitats, from natural to domestic. Moreover, its anthropophily, combined with its ability to colonize urban and periurban areas, amplifies the risk of arbovirus transmission. North Africa appears to be dominated by *Ae. albopictus*, except for Sudan and Egypt, where the presence of *Ae. aegypti* has been reported in recent years [27, 28]. The southern and eastern regions are characterized by the presence of *Ae. aegypti*, apart from South Africa, Tanzania, and Mozambique, where both *Ae. aegypti* and *Ae. albopictus* are present, and Djibouti, where only the presence of *Ae. albopictus* has been documented [28]. The central region is unique for cocirculation of *Ae. aegypti* and *Ae albopictus* in almost all countries, where the expansion of *Ae. albopictus* is gradually reducing the presence of *Ae. aegypti* in anthropized habitats, apart from Equatorial Guinea and Sao Tome and Principe, where only *Ae. albopictus* has been reported [27, 29, 30]. The West African region is known for the predominance of *Ae. aegypti*, although in recent years the invasion of *Ae. albopictus* has been observed in some countries in the region: Nigeria, Mauritania, Côte d'Ivoire, Ghana, Benin, and Mali [26, 27, 30]. However, this introduction into central and western regions raises concerns that *Ae. albopictus* may eventually eliminate *Ae. aegypti* due to its ecological plasticity and invasiveness, which have already been observed elsewhere [28, 30]. In addition, this spatial distribution influences the epidemiological landscape, as the two species may differ in their ability to transmit specific arboviruses.

The distribution of arboviruses transmitted by *Aedes* mosquitoes closely reflects the distribution of their vectors with significant geographical variations. Dengue fever remains endemic and cyclically active in West and East Africa, while CHIKV has caused major epidemics, notably recent outbreaks in Kenya in 2025 [31, 32]. Despite vaccine efficacy, transmission of YFV persists in West and Central Africa, and ZIKV circulates at low levels in several African regions, particularly in the western and central parts of the continent [27, 33]. Although the northern region seems to be relatively unaffected by the four viruses, imported and autochthonous cases of DENV have been reported in Algeria and Egypt from 2015 [28]. The absence of reported cases of CHIKV, ZIKV, and YFV in the region remains ambiguous. It is important to note the co-circulation of several arboviruses in geographical areas [34], which increases the risk of co-infection in humans if the vector present is competent.

Vector competence is the intrinsic ability of mosquitoes to acquire, replicate, and transmit the pathogen to the naïve vertebrate host [1]. It reflects combination of mosquito genetic factors, viral genotype, and environmental conditions. It is generally measured by several parameters, including infection, dissemination, and transmission rates [1]. It is important to note that vector competence varies considerably from one geographic population to another and such variation cannot be fully explained by mosquito genetics or viral genotype. One of the factors contributing to it is the composition of mosquito microbiota, which is increasingly recognized as a determinant of vector competence [19].

The microbiota of *Aedes aegypti* is a complex microbial community composed of various microorganisms occupying specific niches in the host. The bacterial component is consistently dominated by the phylum Proteobacteria, and the viral component by endogenous viruses called insect-specific viruses (ISVs) [35, 36]. Although a core microbiota seems to exist, specific taxa and their relative abundance differ depending on developmental stage, environmental conditions, and geographic origin, hence the concept of pan-microbiota [19]. Thus, in contrast to the core microbiota, the pan-microbiota constitutes the variable or transient microbiota induced by environmental influences. Regardless of stable or transient notion, microbiota depends on complex interactions between host biology, environment and other ecological factors.

- *Host factors and feeding behavior*
  Different mosquito species present unique microbiota profiles, which can be attributed to their evolutionary histories and ecological niches. For example, *Aedes* species may harbor different microbial communities than *Culex* or *Anopheles* species.
  Mosquito genetics and mosquito developmental stages are likely to play a role in the composition of microbiota by determining the ability of mosquitoes to host and maintain certain species of microorganisms. The role of mosquito genetics is still debated. It has been shown that populations of genetically different mosquitoes from six countries raised under the same laboratory conditions carried a similar microbiota, suggesting that within the same species, genetics does not have a strong influence on microbial composition [37]. However, as laboratory colonies were used, it cannot be excluded that this similarity in the composition of the microbiota results from selection due to similar mosquito breeding techniques. In *Ae. aegypti,* the number of bacterial operational taxonomic units (OTUs, cluster of highly similar genetic sequences that represent one or a few closely-related taxa) in newly emerged adults may be two times lower than that in larvae [38]. It suggests that larvae lose part of their bacterial microbiota during metamorphosis, most likely through the meconium [39]. However, some bacteria are maintained from larvae to adults, such as the genera *Acinetobacter, Bacillus, Chryseobacterium, Enterobacter, Pseudomonas, Serratia*, and *Staphylococcus* [19, 38]. During its life, the mosquito consumes several meals (sugar and blood). The type and source of the meal can considerably influence the intestinal microbiota [20, 40]. It has been shown that microbial diversity is slightly reduced in mosquitoes fed on sugar or blood. The microbial diversity could be restored to the same level as that of newly emerged adults 7 days after a blood meal [40]. Similarly, *Ae. aegypti* females fed sugar or blood (chicken, rabbit, or a mixture of chicken and rabbit) presented distinct clusters of gut bacterial communities depending on the source of blood [41]. Furthermore, some bacterial taxa were significantly associated with specific treatments. For example, *Pseudomonas* spp. and *Stenotrophomonas* spp. were significantly associated with mosquitoes that fed on chicken blood. *Rahnella* spp. and *Actinotignum* spp. were associated with mosquitoes that fed on rabbit blood, whereas unclassified *Acetobacteriaceae* were indicator species for mosquitoes that fed on a mixture of chicken and rabbit blood. While no species was significantly associated with sugar-fed mosquitoes, taxa such as *Aeromonas* spp.*, Serratia* spp., *Burkholderiaceae*, and *Enterobacteriaceae* were associated with several treatments [41]. In addition, mixed blood meals can have a synergistic effect on OTU richness and the Shannon diversity index [41], suggesting that mixed meals can be used to obtain specific bacteriome profiles. In West Africa, the subspecies *Aaa* (anthropophilic) and *Aaf* (zoophilic) cohabit [42, 43] and can feed on several hosts; it is therefore obvious that these mixtures of meals from various sources can consequently modify the microbiota by selecting a particular microbial community associated with a particular VC profile. Given that only females are hematophagous and that the blood meal induces a modification of the mosquito microbiota, sex is also a factor influencing the composition of the microbiota of mosquitoes [44, 45]. In addition, females are likely to be associated with hemolytic bacteria to facilitate the digestion of the blood meal [14].
- *Environmental factors*
  The ambient environment plays a pivotal role in shaping the microbiota of mosquitoes. Ecological factors, such as temperature, humidity, and the availability of organic matter, can influence the microbial communities associated with different insect species. Temperature during development affects the thermal tolerance and microbial composition of *Drosophila melanogaster* flies. Therefore, low temperatures (13 °C) favor *Wolbachia* within the microbiota and better resistance to cold, whereas high temperatures (31 °C) promote *Acetobacter* and heat tolerance in flies [46]. Similarly, in the female sandfly *Lutzomyia longipalpis*, microbiota structure variations were observed according to the temperature at which the hosts were maintained. These variations were most pronounced between 29 °C and 33 °C, with certain bacterial genera significantly more abundant than others [47]. In *Ae. aegypti* mosquitoes, the exposure of larvae to two distinct temperature regimes led to significant differences in the intestinal microbiota of the mosquitoes. An increase in microbial richness was associated with an increase in temperature. Thus, the bacterial species *Bacillus subtilis*, *Acidovorax citrulli*, and *Pseudomonas aeruginosa* are particularly enriched when the temperature increases, unlike the *Dermacoccus* genus [48]. Similarly, a decrease in temperature affects the growth of microbial populations in *Ae. albopictus*. Therefore, the shift from 28 °C to 18 °C was associated with an increase in the genera *Elizabethkingia* sp. and *Chryseobacterium* sp. to the detriment of *Klebsiella oxytoca* [49]. In *Ae. albopictus*, the bacterial microbiota of mosquitoes reared at 28 °C and held at 20 °C after a blood meal was characterized by greater bacterial diversity, with a predominance of *Sphingomonadaceae, Rhizobiaceae*, and *Micrococcaceae*, whereas mosquitoes raised under the same conditions but placed at 28 °C after blood feeding had a relatively undiversified microbiota dominated by *Asaia* [50].
  In contrast, Bellone et al. [51] reported that the diversity and dominant genera of the mosquito bacterial microbiota of *Ae. albopictus* raised under the same conditions and placed at different temperatures after infection with CHIKV significantly varied depending on the temperature and transmission profile [50]. In addition, maintaining mosquitoes at 28 °C following CHIKV infection significantly reduced *Wolbachia* abundance, whereas *Serratia* abundance increased, resulting in increased viral infection in mosquitoes [50]. As *Serratia* has been shown to promote viral infection, whereas *Wolbachia* compromises viral infection, this study demonstrated that temperature changes could significantly impact the dynamics of the microbiota, and in turn, VC.
  Although the literature on the impact of temperature on the microbiota is not uniform, it seems clear that temperature fluctuations affect the richness and/or diversity of the host microbiota. Given that an increasing number of the bacterial species or genera associated with these temperature-dependent modulations are also affected during viral infections (e.g., *Elizabethkingia*) [51, 52], improving our understanding of the impact of temperature on the intestinal microbiota, and in a spinoff effect, on the VC of mosquitoes, is essential.
  Moreover, relative humidity also affects the mosquito microbiota; a humidity gradient influences the relative abundance of several bacterial symbionts present in the microbiota of *Ae. albopictus* [53]. The microbiota composition of mosquitoes varies across the entire landscape. Regardless of whether the environment is hot and dry or cool and humid, certain bacterial genera are relatively more abundant [53]. Similarly, the presence of organic matter, such as leaf litter, at breeding sites has a significant effect on the composition of mosquito bacterial communities [54].
  Geographical and seasonal variations also affect the microbiota composition of insects. For example, in Cameroon, significant variations in the bacterial composition of *An. coluzzii* were observed between the dry and rainy seasons. Clusters of symbionts, such as *Asaia, Serratia, Enterobacter*, and *Pseudomonas*, were found to depend on the season and the locality. However, certain genera were found to be independently associated with both the season and the locality [55].
  Overall, environmental factors such as temperature, relative humidity, organic matter, and geographical and seasonal variations play key roles in structuring the microbiota of mosquitoes.
- *Microbial interactions*
  Prior to interacting with disease-causing microorganisms that may colonize the vector host, the microbiota interacts with itself. In other words, the microbial communities interact with each other in a complex way within the host through mechanisms of competition and cooperation. The result of these interactions is a rich and diverse microbiota that, in a subtle balance, performs various functions in the host. In many hosts, competition results in resistance to colonization by nonresident microorganisms. It combines resistance to initial infection, improved tolerance to established infection, and elimination of infection. Resistance results from direct and indirect mechanisms such as invasion of the ecological niche, competition for resources and space, secretion of antimicrobial molecules, competitive exclusion, and stimulation of the host immune system [56]. For example, resistance to colonization has been observed in *Anopheles*, with the inhibition of vertical transmission of *Wolbachia* in the presence of *Asaia* in the microbiota of *An. gambiae* and *An. stephensi* [57, 58]. Similarly, the inhibition of *Asaia* by *Pseudomonas* and *Pantoea* in mosquitoes (*Aedes*, *Anopheles*, and *Culex*) confirms the competition between different bacterial communities that make up the host microbiota [59].
  Conversely, cooperation enables microbes to maximize the exploitation of resources, ensure their survival, and reinforce the insect’s physiology. This is illustrated by the yeast‒endobacteria system in mosquitoes, where the yeast *Wickerhamomyces anomalus* hosts certain bacteria from the intestinal microbiota in the context of stress while providing metabolic and/or antimicrobial support to the host [60, 61].
- *Anthropogenic factors*
  Environmental degradation, particularly through chemical pollution (pesticides, insecticides, heavy metals, etc.), is likely to modify the dynamics of microbial communities present in the environment, and consequently, in larval habitats and plants, natural sources of sugar [62]. The accumulation of these pollutants in the environment can influence the interactions between the microbiota and mosquitoes, thus affecting their development and their ability to transmit pathogens. These changes are likely to have a greater impact in Africa, where environmental degradation is on the rise, coupled with increasing resistance of mosquitoes to insecticides. It has been shown that insecticide-resistant mosquitoes harbor specific microbiota [63, 64]. The same is true for urbanization or changes in land use, which are likely to modify the availability of aquatic ecosystems where mosquitoes breed [65]. In both situations, a change in the microenvironments where vectors evolve will impact mosquito vector capacity in a domino effect, either by changing VC, impacting survival, or creating conditions conducive to increased host‒vector contact. Under natural conditions, these factors are more likely to be intertwined, and the magnitude of the expansion of arboviral diseases observed recently results from the impact of global changes, urbanization, and human mobility.

### Microbiota-pathogen interactions in *Aedes aegypti*

**Figure 2:**
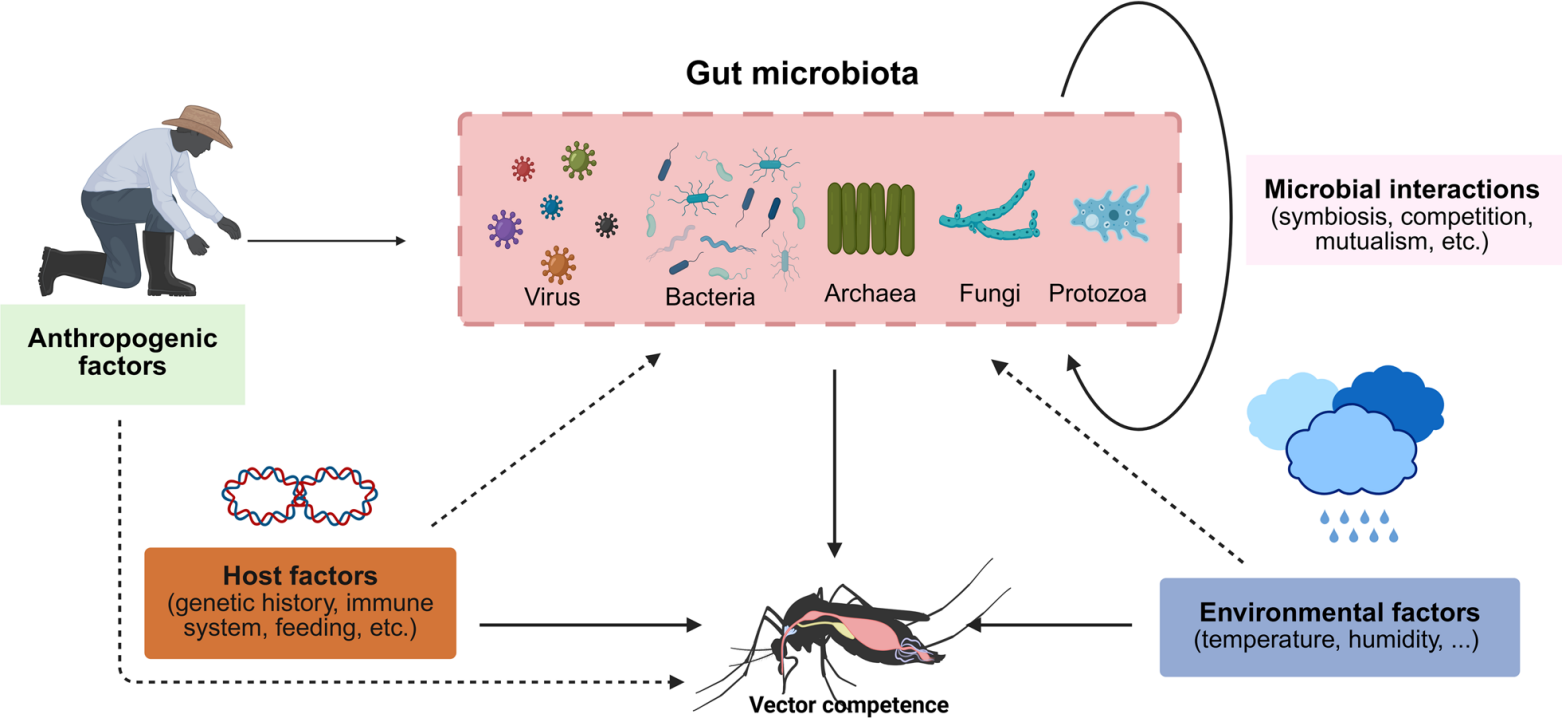
Factors determining mosquito vector competence. Created in BioRender. DOKPOMIWA, H. (2025) https://BioRender.com/7geyd1s

The transmission of arboviruses first requires the infection of a female mosquito during a blood meal on a viremic host. This is followed by replication of the virus within the mosquito, and finally, transmission of the virus to a susceptible host during the next blood meal [1, 66]. During these successive steps, the virus must escape from host immune responses, cross the various epithelial barriers associated with the midgut and salivary glands [67], and interact with the resident microbial flora colonizing different organs, particularly the midgut. Passing successfully through these different steps is an indication of the ability of the vector to transmit the virus. The VC for an arbovirus thus depends not only on factors intrinsic to mosquitoes, but also on other significant extrinsic factors (Fig. 2).

Although the emphasis is often oriented toward the role of microbiota in lowering virus transmission, it is important to stress that microbiota can also increase the ability of the vector to transmit a virus. The microbiota may reduce vector competence through three ways: directly by the synthesis of antiviral molecules and indirectly by activation of the mosquito’s innate immune system or creation of unfavorable conditions for viral replication [68]. Considering the large number of nonpathogenic ISVs and their genetic proximity with arboviruses, it has been shown that ISVs can modify the susceptibility of mosquitoes to arboviruses [69–71]. While most artificial ISV infections have a negative impact on arbovirus transmission, natural infections often have positive associations [72]. The VC of *Ae. aegypti* is complex and far from completely understood, but the mosquito microbiota reveals a significant role.

- *Microbiota aims to reduce arbovirus transmission by Ae. aegypti*
  The *Ae. aegypti* microbiota includes a variety of bacteria. *Wolbachia pipientis* is one of them that has been shown to reduce or even inhibit the transmission of arboviruses in *Ae. aegypti*. While *Wolbachia* is not naturally found in *Ae. aegypti*, introducing these strains through transfection is strongly associated with reduced viral susceptibility to DENV, ZIKV, and CHIKV [73–78]. The use of *Wolbachia*-based strategies in the control of *Ae. aegypti* in Australia, Brazil, and Malaysia has highlighted the potential of using the microbiota as a vector control strategy [79–83]. Therefore, the potential of other members of microbiota to disrupt arbovirus transmission by *Ae. aegypti* should be further explored. To date, *Proteus* spp., *Chromobacterium* sp. Panama (Csp_P), and *Rosenbergiella* are some bacteria from the *Ae. aegypti* microbiota that have shown antipathogenic activity against arboviruses in experimental studies. Indeed, Ramirez et al. (2012) reported that *Proteus* sp. (Prsp_P) and *Chromobacterium* sp. (Csp_P), which were isolated from *Ae. aegypti* populations in Panama, reduced susceptibility to DENV infection. This was observed when each bacterium was reintroduced into mosquitoes first exposed to antibiotics [84, 85]. In addition, it has been shown that *Chromobacterium* sp. (Csp_P) also has an entomopathogenic effect on larvae and adults when exposed to bacterial isolates [85]. Moreover, when raised in the presence of Enterobacteriaceae isolates, gnotobiotic mosquitoes are more permissive to DENV, unlike gnotobiotic mosquitoes raised in the presence of *Salmonella* isolates [16]; Enterobacteriaceae can interfere with DENV transmission. Moreover, the bacterium *Rosenbergiella* associated with plant nectar was detected in the midgut of *Ae. aegypti* mosquitoes collected in areas with a low incidence of dengue, unlike the microbiota of mosquitoes from dengue endemic areas [86]. The *Rosenbergiella YN46* strain experimentally introduced into the microbiota of *Ae. aegypti* provided resistance to DENV and ZIKV. In addition, the bacteria are stable in the mosquito midgut and undergo trans-stadial transmission [86].
  While several studies have focused primarily on bacteria, the presence of viruses in the microbiota of *Ae. aegypti* has also been explored. Insect-specific viruses (ISVs) and some bacteria-associated viruses (bacteriophages) have been shown to influence the susceptibility of *Ae. aegypti* to DENV. Indeed, a mutual increase was observed in Aa20 cells of *Ae. aegypti* between the cell fusion agent virus (CFAV) and DENV [87]. However, Baidaliuk et al. [69] demonstrated that pre-infection with CFAV reduced DENV and ZIKV titers in *Ae. aegypti* [69]. These results highlight the gap between in vitro and in vivo systems, although the genotype of the virus, the genetics of the mosquito, and the existence of certain confounding factors may also contribute to this difference.
- *Microbiota aims to increase arbovirus transmission by Ae. aegypti*
  Unlike the symbionts previously described, there are also symbionts that have positive interactions with arboviruses in *Ae. aegypti*. Intuitively, they would not be good candidates for arbovirus control or vector control, but it remains essential for understanding transmission mechanisms, anticipating ecological risks, and designing long-term interventions. In addition, exploiting these microbial communities in a strategy to block the pathogen or reduce survival, two key elements of vector capacity, would be interesting. *Serratia marcescens* and *Serratia odorifera* have been described as promoting the susceptibility of *Ae. aegypti* to DENV, increasing the ability of the vector to transmit the virus [88, 89].
  Similarly, the ISVs Phasi-Charoen-like virus (PCLV) and Humaita Tubiacanga virus (HTV) have been shown to facilitate ZIKV transmission in *Ae. aegypti* [71]. These two ISVs are highly abundant in *Ae. aegypti*. These ISVs cocirculated with DENV in *Ae. aegypti* populations collected in dengue-endemic areas [71]. Although the data did not reveal a significant difference in the transmission of DENV by mosquitoes carrying or not carrying the two ISVs, the presence of these ISVs was correlated with the shortening of the extrinsic incubation period (EIP) of ZIKV [71]. These findings indicate that mosquitoes carrying these ISVs need less time to transmit ZIKV efficiently. In addition to bacteria and viruses, other microorganisms such as fungi are likely to influence the VC of *Ae. aegypti*; infection of *Ae. aegypti* with the Puerto Rican fungus *Talaromyces* promotes infection of mosquitoes with DENV [90].
- *…with a focus on African Aedes*
  Only a few studies have explored the microbiota of African *Ae. aegypti* populations. To enrich the database, in this chapter, we will include studies that have investigated the microbiota of African *Ae. albopictus* populations. However, most studies describe the diversity of the microbiota without establishing a correlation between the microbial community and the transmission of arboviruses (Additional file 1: Table S1).

In Africa, *Ae. aegypti* has two subspecies, *Ae. aegypti aegypti* (*Aaa*) and *Ae. aegypti formosus* (*Aaf*), which differ in their morphology, ecology, and genetics [24]. Recent studies indicate that this distinction is no longer clear in African populations of *Ae. aegypti*. In Kenya and probably East Africa, evidence suggests that the two subspecies colonize artificial and natural shelters in sympatry [91, 92]. In West Africa, the differentiation between these subspecies is gradually becoming blurred, and differences based on the presence or absence of white scales on the first tergite may be insufficient [42]. Indeed, over several generations from females of either of the two forms, heterogeneous families comprising both forms are obtained. The offspring of a couple belonging to a homogeneous subspecies may exhibit characteristics corresponding to another subspecies. This could suggest an insufficient number of identification markers or phenotypic plasticity. Furthermore, it has been demonstrated in Senegal that *Aaa* and *Aaf* circulate in urban areas, feeding mainly on humans [93]. This may be due to progressive urbanization and the gradual destruction of forests, leading to *Aaf* adaptation to the urban environment. However, the microbiota of African *Ae. aegypti* may differ from one region to another on the continent. This is particularly because different egg-laying habits, varying blood feeding preferences, and distinct environments could influence the microbial composition of these mosquitoes. Given the intraspecific variability observed in West Africa, it is possible that populations in this region present fewer microbial differences. The identification of a typical ISV “formosus virus” from an *Ae. aegypti formosus* colony originating in Uganda (East Africa) that has not been described in *Aaa* by Parry et al., [94] suggests that once two morphotypes are distinct, the subspecies may conserve specificities in their microbial profile. It also indicates that the microbiota of East African *Ae. aegypti* may differ from that observed in other regions of the continent if the behavior of the vectors is different.

An analysis of African *Ae. aegypti* behavior revealed that the choice of oviposition site differed from that of non-African populations [95]. As the larval environment ultimately influences the mosquito microbiota and both natural and artificial breeding sites do not contain the same microbiota, these findings suggest that African populations may have a unique microbiota compared with that of global populations. To investigate this, we compared the microbiota composition of African *Ae. aegypti* with that of other continents (Additional file 2: Table S3). Although our data are limited, this brief analysis revealed that the African *Ae. aegypti* microbiota differs somewhat from that of other populations around the world. While ISVs found elsewhere, such as Phasi-Charoen-like virus, cell-fusing agent virus, Humaita Tubiacanga virus, and *Aedes* Anphevirus, are also present, certain ISVs are currently specific to African populations, such as the Fako virus and Formosus virus [69, 71, 94, 96, 97]. With respect to the bacterial and fungal microbiota, we do not have sufficient data from Africa to perform unbiased analysis. However, typical bacterial profiles can be observed on other continents. There is a wide distribution of *Asaia, Serratia*, and *Pseudomonas* within the microbiota of Asian and American populations, whereas *Wolbachia*, *Bacillus*, and *Enterobacter* predominate within the bacterial microbiota of Asian *Ae. aegypti* [37, 98–107].

Although patchy, available data from Africa regarding ecological and behavioral differences suggest that African populations of *Ae. aegypti* carry unique microbiota, potentially distinct from those observed on other continents. This highlights the importance of geographical origin of vector microbiota and the necessity of exploring the microbiota of African *Ae. aegypti*.

### Mechanisms underlying *Ae. aegypti* VC modulation by the gut microbiota

During arbovirus infection, the mosquito microbiota can mediate the success or failure of infection through various mechanisms. Although some of these mechanisms are unclear, several hypotheses have been proposed, and in some situations, interactions between the mosquito microbiota and the arbovirus are bidirectional [108]. The microbiota interacts with the pathogen to prevent its maintenance, replication, or transmission, and the pathogenic virus can also influence the composition of the microbiota. Some studies have demonstrated the influence of DENV or ZIKV on the bacterial and nonpathogenic microbiota of *Ae. aegypti* [84, 108]. In parallel, the microbiota of *Ae. aegypti* influences the transmission of arboviruses by modulating immune responses, metabolism, and development or by establishing competition for resources.

A significant reduction in the transmission of DENV, ZIKV, CHIKV, and yellow fever virus (YFV) in mosquitoes infected with the bacteria *Wolbachia* is associated mainly with an increased mosquito immune response [78, 109, 110]. Several hypotheses have been proposed, and this may be due to the activation of the immune pathways Toll, Janus kinases/signal transducers and activators of transcription (JAK/STAT), immune deficiency (IMD), and RNAi following the production of antimicrobial peptides (AMPs) or reactive oxygen species (ROS) [109]. Similarly, *Wolbachia* can compete for resources by sequestering cholesterol or by inducing the production of microRNAs (miRNAs), suppressing the expression of essential genes during the methylation of the viral genome [111, 112]. These mechanisms are far from able to induce alone the pathogen-blocking phenotype observed in mosquitoes transinfected with *Wolbachia*; it is thought that this phenomenon is the result of several mechanisms that take place in an interlocking manner [6]. The microbiota can also act by competing with pathogens for resources and ecological niches within the host [113]. Beneficial bacteria can inhibit the growth of viruses and other pathogens via mechanisms such as the production of antimicrobial metabolites or the simultaneous colonization of biological spaces [113]. This competition can reduce the mosquito viral load and consequently its VC. Similarly, bacteria in microbiota also influences the development and survival of *Ae. aegypti,* with consequences for its role as vectors [114]. Healthy microbiota can contribute to better larval growth and optimal development, thus increasing the longevity of adult mosquitoes. A prolonged lifespan can also be correlated with an increase in opportunities for pathogen transmission.

Finally, microbiota plays a key role in the metabolism of nutrients and compounds present in the blood, which can influence the reproduction and life traits of mosquitoes. For example, some microorganisms can breakdown specific nutrients into metabolites used for mosquito development. *Serratia marcescens* promotes arbovirus infection by degrading mucins bound to the intestinal membrane of *Ae. aegypti* and releasing the protein Sm, which decreases the natural protection of the intestinal mucus [89].

Similarly, the increased susceptibility to DENV infection observed in *Ae. aegypti* upon infection with the fungus *Talaromyces* sp. is based on the suppression of the expression of digestive enzymes in the midgut of *Aedes* mosquitoes [90].

### Global changes and mosquito: epidemiological challenges in Africa

Global warming has a strong impact on our environment, particularly by creating conditions that favor the spread of vectors. These changes include extreme climatic events (heat waves, hurricanes, and floods), rainfall, air quality changes, sea level rise, and temperature changes [115]. Ambient temperature plays a central role in the growth and behavior of mosquitoes. For example, an increase in temperature can affect the presence of certain bacteria, viruses, or yeasts (nonpathogenic to humans), increasing the likelihood of transmitting arboviruses.

Around the world, particularly in Africa, the increase in temperature caused by global climate change has promoted not only the growth of *Aedes* mosquitoes, but also their expansion into new geographical areas [115]. This increases contacts between mosquitoes and people, who are often naive to multiple arboviruses. A crucial aspect related to the increase in temperature concerns the EIP of the virus in mosquitoes, which is often used as an indicator of VC [116]. This corresponds to the time required to ensure that the virus replicates and reaches the mosquito saliva, making it infectious, after an infected blood meal. Numerous studies and reviews have shown that high temperatures accelerate this incubation period, thereby shortening the EIP [1, 116, 117]. Notably, if the EIP is short, the epidemic potential of the virus will increase. Although the relationship between temperature and arbovirus transmission is nonlinear [118], in an African context, the combination of an accelerated transmission cycle and increased *Ae. aegypti* density is likely to make the region more conducive to arbovirus transmission than malaria transmission [119]. Mordecai et al. [119] provide an extensive explanation of this in their personal view, relying on climatic and epidemiological models. Consequently, the threat posed by arbovirus pandemics could surpass that posed by malaria, necessitating the adaptation of surveillance and control strategies [119]. The thermal optima for transmission of malaria parasites by *Anopheles* mosquitoes peak at 25 °C, whereas arbovirus transmission by *Ae. aegypti* peaks at 29 °C.

The situation becomes even more serious when we consider the joint circulation of several closely related viruses transmitted by the same vector, *Ae. aegypti*. Initially, the joint circulation of two or three *Aedes*-borne arboviruses was observed in humans, particularly in areas where these viruses share the same distribution range. Examples include Thailand between 2018 and 2020, where there were coinfections involving CHIKV, DENV, and ZIKV; an unprecedented case of YFV and DENV-2 coinfection in Angola in 2016; and multiple cases of coinfection involving DENV, YFV, and WNV in Ogbomoso, Nigeria, in 2017 [120–122]. Given that coinfections exist in humans, this phenomenon can also be observed in mosquitoes. Unfortunately, this is rarely observed in the field. Nevertheless, coinfection (either simultaneous or sequential) can be reproduced in mosquitoes in laboratory conditions. Depending on the combinations, coinfection can be expected to promote or inhibit the transmission of one or both viruses, cause competition between them, or have no effect on transmission [123]. Rückert et al. reported that *Ae. aegypti* mosquitoes can be infected with different combinations of viruses, such as CHIKV/DENV-2, CHIKV/ZIKV, and DENV-2/ZIKV, and that mosquitoes are able to transmit the viruses in 7 days [124]. However, when mosquitoes were coinfected with CHIKV and ZIKV, the EIP decreased to 3 days, which favored ZIKV transmission, which was not transmitted when the virus was provided alone. This raises the question of variations in the EIP when arboviruses are cocirculating and transmitted by the same vector.

Second, the impact of temperature variations on coinfections should be considered. Each arbovirus has specific temperature requirements for transmission [116, 125]. This raises the question of whether coinfection could alter these requirements, thereby enabling or preventing transmission outside the optimal temperature range. While this issue remains unclear, Terradas et al. [119] reported that temperature affects the viral kinetics of *Ae. aegypti* when it is coinfected with the Mayaro virus and DENV [118].

In summary, climate change affects the landscape of vector-borne diseases by affecting mosquito physiology, microbiota, and VC through temperature variations. This alteration can lead to a shift toward arbovirus transmission, surpassing malaria, due to factors such as *Aedes* mosquito geographical expansion and shortened virus incubation periods. The implications of these changes underscore the necessity of enhancing epidemiological surveillance, adjusting vector control strategies, and persisting with research efforts to comprehend and address the intricate connections exacerbated by accelerating climate change.

## Limitations, research gaps, and perspectives

Vector control based on the exploitation of microbiota is a promising strategy to control mosquito-borne diseases [126]. Despite the importance of understanding the interactions between microbiota and pathogens in *Ae. aegypti*, this review includes some limitations. Insufficient available data limit this review scope to determine whether the microbiota of African populations of *Ae. aegypti* differs significantly from that of populations found beyond African borders, as well as any variations it may exhibit at the regional or country levels. This constraint is due to microbiota of African *Ae. aegypti* being poorly studied compared with that of Europe, Asia, or America, making it unreliable to compare different geographic regions. This bias may therefore hide geographic variations in the composition of microbiota. While some studies have documented insect-specific viruses in *Ae. aegypti*, their global distribution and influence on vector competence remain insufficiently characterized. In addition, fungal and viral components of microbiota remain poorly studied compared with bacterial communities. These gaps in literature limit our ability to present a complete view of how microbial communities is shape in African *Ae. aegypti*.

Another limitation is the lack of studies that simultaneously characterize microbiota composition and assess vector competence profiles in *Ae. aegypti* populations from Africa. While some studies document *Ae. aegypti* microbiota diversity and others examine vector competence for specific pathogens, none establish a correlation between bacterial, viral, or fungal taxa and arbovirus infection, dissemination, or transmission rates. This has limited our ability to establish correlations between microbiota and vector competence in the African context.

These limitations highlight the need for future research on African *Ae. aegypti* populations, integrating microbiota characterization with vector competence assessment while studying the bacterial, viral, and fungal components of the microbiota. These knowledge gaps need to be filled before microbiota-based approach becomes a fully effective antivectorial strategy. Certain key features have already been highlighted, but several questions still need to be addressed. These science-related challenges are summarized in Additional file 1: Table S2.

In addition to these scientific questions, other operational challenges remain. Microbiota-based strategy combines several advantages, including its effectiveness in controlling vector-borne diseases transmitted by *Ae. aegypti*. To address the increasing resistance to insecticides developed by mosquitoes, vector control should be more holistic by combining nonchemical methods with those already available. Given the specificity of the mosquito microbiota to geographical environments [15, 55, 127], it seems more realistic to design strategies on the basis of the microbiota present in mosquitoes of each region or on bacteria that are stably established and have a strong impact on vector transmission. The focus could be placed on regions with high potential for epidemics or regions with risks of endemicity of arboviruses transmitted by *Ae. aegypti*. Strategies using the microbiota will complement other control strategies, and several approaches can be combined. Two approaches can be considered for combined use or to reinforce other existing strategies. The first approach involves compromising viral transmission by mosquitoes. This will require identifying the bacterial species that increase or reduce vector competence in the vector. To better characterize the mechanisms involved, the response leading to the pathogen-blocking phenotype should be optimized. The second approach involves the use of species that significantly impact the development and survival of immature and adult mosquitoes. This approach reduces the density of vectors in a specific environment. Microbiota-based strategies have the advantage of combining several mechanisms at the same time, limiting the occurrence of resistance.

To date, only the use of *Wolbachia* constitutes a microbiota-based vector control technique that has evolved and benefited from larger-scale trials [14]. Although this is a notable advance, its use remains exceptional because the mechanisms supporting the pathogen-blocking phenotype are still poorly understood. Vector control strategies based on microbiota are expensive and require multidisciplinary approaches. This raises the question of its implementation in low- and middle-income countries (LMICs), particularly in Africa. In Africa, which is facing increasing epidemics due to arboviruses, research efforts should be reinforced to obtain decisive information for the implementation of microbiota-based vector control. These needs are clearly illustrated by the implementation of *Wolbachia*-based strategies in Africa. In other regions, this work is well advanced, or better yet, in the phase of large-scale use. To our knowledge, no field trials have been conducted in Africa. Existing studies are limited to laboratory data, such as those conducted in Burkina Faso [128, 129], which are currently progressing toward field trials.

Another limitation is sustainability or stability. Indeed, the implementation of these strategies will have to provide proof of their effectiveness in heterogeneous natural environments, and at the same time, guarantee that the changes made to the microbiota promote lasting effects on vector competence or on the development/survival of mosquitoes. Finally, these strategies should receive the acceptance of local communities and require their implications. It is therefore necessary to conduct in-depth and multidisciplinary studies to provide evidence that this strategy is safe for the environment and living organisms, including humans.

## Conclusions

In Africa, *Ae. aegypti* and *Ae. albopictus* are present even if their distribution is dynamic and almost overlaps with the distribution of viruses they transmit. The exploration of *Ae. aegypti* microbiota is promising for improving our knowledge of the different microbial communities that coexist within the mosquito. Similarly, the complex relationship between the microbiota of *Ae. aegypti* and its vector competence opens promising avenues for vector control and arbovirus management. This area of research has attracted particular interest in recent years because the microbiota impacts mosquito life traits (reproduction, diet, fitness, etc.) and the intrinsic ability of mosquitoes to transmit arboviruses. However, microbiota varies according to many factors, including those related to the mosquito itself, human activities, and environmental conditions. To date, several bacterial, viral, and fungal targets that either promote or compromise viral transmission within the vector have been identified. Within African populations of *Ae. aegypti*, despite limited research on the subject, some microbial targets have also been described, but their direct involvement in a viral transmission phenotype has not yet been demonstrated. Despite the nonlinearity of the microbiota‒vector competence interaction, changes in the microbiota indirectly affect its vector competence. Thus, at the African scale, global changes and urbanization, which are both liable to shape the microbiota of vectors, present further epidemiological challenges. These changes could increase the distribution of *Ae. aegypti* and disrupt the epidemiology of vector-borne diseases. These potential threats highlight the need for further research into the development of vector control tools adapted to the specificities of Africa. Overall, microbiota-based approaches offer compelling advantages, the most emblematic example being the use of *Wolbachia* in dengue control. However, we are far from transcribing the existing scientific data on the microbiota into operational control tools owing to some operational and scientific challenges. These include the need for a deeper knowledge of the interactions between the microbiota and its host, as well as ecological variability, acquisition, and persistence mechanisms of the microbiota, etc. As these technologies are costly and require expertise and infrastructure that are limited in LMIC and population acceptance, it will be essential to prioritize in-depth studies of mosquito microbiota in the African context. These investigations will provide a comprehensive overview of the specific opportunities and challenges within each region, enabling effective optimization and acceptance of future interventions.

## Supplementary Information

Additional file 1.

Additional file 2.

## Data Availability

Data supporting the main conclusions of this study are included in the manuscript.

## Abbreviations

Aaa: *Aedes aegypti aegypti*
Aaf: *Aedes aegypti formosus*
*Ae. aegypti*: *Aedes aegypti*
*Ae. albopictus*: *Aedes albopictus*
*An. coluzzii*: *Anopheles coluzzii*
*An. stephensi*: *Anopheles stephensi*
AMP: Antimicrobial peptide
CFAV: Cell fusion agent
CHIKV: Chikungunya virus
Csp_P: *Chromobacterium* sp. from Panama
DENV: Dengue virus
EIP: Extrinsic incubation period
HTV: Humaita Tubiacanga virus
IMD: Immune deficiency
ISV: Insect-specific virus
JAK/STAT: Janus kinases/signal transducers and activators of transcription
LMICs: Low- and middle-income countries
miRNA: MicroRNA
OTU: Operational taxonomic unit
PCLV: Phasi-Charoen-like virus
Prsp_P: *Proteus* sp. from Panama
RNAi: RNA interference
sp.: Species
spp.: Species plural
VC: Vector competence
WNV: West Nile virus
YFV: Yellow fever virus
ZIKV: Zika virus
