# Supplementary material for: Unlocking new frontiers in vector control strategies using Aedes aegypti microbiota
Source: Parasit Vectors. 2026 May 5;19:260. doi: 10.1186/s13071-026-07304-5 (PMC13289545; doi:10.1186/s13071-026-07304-5)
Supplement: Supplementary file 1 — Additional file 1. [file 13071_2026_7304_MOESM1_ESM.docx]

Table S1 Microbiota target identified in African *Aedes* mosquito

| Country | Host | Sample type | Target | Linked with transmission phenotype | Microbial type | References |
| --- | --- | --- | --- | --- | --- | --- |
| Gabon | *Ae. aegypti* | Lab colony | *Pseudoalteromonas* | No | Bacterial | [1] |
| Gabon | *Ae. aegypti* | Lab colony | *Pseudomonas* | No | Bacterial |  |
| Gabon | *Ae. aegypti* | Lab colony | *Luteibacter* | No | Bacterial |  |
| Gabon | *Ae. aegypti* | Lab colony | *Rhodanobacter* | No | Bacterial |  |
| Gabon | *Ae. aegypti* | Lab colony | *Enterobacter* | No | Bacterial |  |
| Uganda | *Ae. aegypti* | Lab colony | *Pseudomonas* | No | Bacterial |  |
| Uganda | *Ae. aegypti* | Lab colony | *Pseudoalteromonas* | No | Bacterial |  |
| Uganda | *Ae. aegypti* | Lab colony | *Luteibacter* | No | Bacterial |  |
| Uganda | *Ae. aegypti* | Lab colony | *Rhodanobacter* | No | Bacterial |  |
| Uganda | *Ae. aegypti* | Lab colony | *Marinomonas* | No | Bacterial |  |
| Réunion | *Ae. albopictus* | Lab colony | *Sphingomonas* | No | Bacterial | [2] |
| Réunion | *Ae. albopictus* | Lab colony | *Chryseobacterium* | No | Bacterial |  |
| Réunion | *Ae. albopictus* | Lab colony | *Dysgonomonas* | No | Bacterial |  |
| Réunion | *Ae. albopictus* | Lab colony | *Corynebacterium* | No | Bacterial |  |
| Réunion | *Ae. albopictus* | Lab colony | *Massilia* | No | Bacterial |  |
| Réunion | *Ae. albopictus* | Lab colony | *Cloacibacterium* | No | Bacterial |  |
| Réunion | *Ae. albopictus* | Lab colony | *Paracoccus* | No | Bacterial |  |
| Réunion | *Ae. albopictus* | Lab colony | *Micropruina* | No | Bacterial |  |
| Réunion | *Ae. albopictus* | Lab colony | *Pelomonas* | No | Bacterial |  |
| Réunion | *Ae. albopictus* | Lab colony | *Enhydrobacter* | No | Bacterial |  |
| Réunion | *Ae. albopictus* | Lab colony | *Wolbachia* | No | Bacterial | [3] |
| Kenya | *Ae. aegypti* | Field mosquitoes | Cell fusing agent virus | No | ISV | [4] |
| Madagascar | *Ae. aegypti* | Field mosquitoes | Guadeloupe Culex *rhabdovirus* | No | ISV | [5] |
| Madagascar | *Ae. aegypti* | Field mosquitoes | Grenada mosquito *rhabdovirus* 1 | No | ISV |  |
| Madagascar | *Ae. albopictus* | Field mosquitoes | Xiang Yun picorna-like virus 4 | No | ISV |  |
| Madagascar | *Ae. albopictus* | Field mosquitoes | Guadeloupe Culex *rhabdovirus* | No | ISV |  |
| Madagascar | *Ae. albopictus* | Field mosquitoes | Grenada mosquito *rhabdovirus* 1 | No | ISV |  |
| Madagascar | *Ae. aegypti* | Field mosquitoes | Xiang Yun picorna-like virus 4 | No | ISV |  |
| Kenya | *Ae. aegypti* | Field mosquitoes | *Aedes-flavivirus* | No | ISV | [6] |
| Kenya | *Ae. aegypti* | Field mosquitoes | Cell fusing agent virus | No | ISV |  |
| South Africa | *Ae. aegypti* | Field mosquitoes | Cell fusing agent virus | No | ISV | [7] |
| Kenya | *Ae. aegypti* | Field mosquitoes | Cell fusing agent virus | No | ISV | [8] |
| Nigeria | *Ae. aegypti* | Field mosquitoes | *Ae. aegypti* totivirus | No | ISV | [9] |
| Nigeria | *Ae. albopictus* | Field mosquitoes | *Ae. aegypti* totivirus | No | ISV |  |
| Nigeria | *Ae. aegypti* | Field mosquitoes | Cell fusing agent virus | No | ISV |  |
| Nigeria | *Ae. aegypti* | Field mosquitoes | Chaq-Like virus | No | ISV |  |
| Nigeria | *Ae. albopictus* | Field mosquitoes | Chaq-Like virus | No | ISV |  |
| Nigeria | *Ae. aegypti* | Field mosquitoes | Fako virus | No | ISV |  |
| Nigeria | *Ae. aegypti* | Field mosquitoes | Phasi Charoen-like virus | No | ISV |  |
| Nigeria | *Ae. albopictus* | Field mosquitoes | Phasi Charoen-like virus | No | ISV |  |
| Nigeria | *Ae. aegypti* | Field mosquitoes | Tesano Aedes Virus | No | ISV |  |
| Nigeria | *Ae. albopictus* | Field mosquitoes | Verdadero virus | No | ISV |  |
| Senegal | *Ae. aegypti* | Field mosquitoes | Aslam narnavirus | No | ISV | [10] |
| Gabon | *Ae. albopictus* | Field mosquitoes | Guangzhou sobemo-like virus | No | ISV |  |
| Senegal | *Ae. aegypti* | Field mosquitoes | Humaita-Tubiacanga virus | Yes | ISV |  |
| Senegal | *Ae. aegypti* | Field mosquitoes | Phasi Charoen-like virus | Yes | ISV |  |
| Réunion | *Ae. albopictus* | Field mosquitoes | Kamiti river virus | No | ISV | [11] |
| Réunion | *Ae. albopictus* | Field mosquitoes | Cell fusing agent virus | No | ISV |  |
| Réunion | *Ae. albopictus* | Field mosquitoes | *Aedes*-flavivirus | No | ISV |  |
| Gabon | *Ae. aegypti* | Lab colony | *Aedes-*anphevirus | Yes | ISV | [12] |
| Kenya | *Ae. aegypti* | Lab colony | *Aedes* anphevirus | Yes | ISV |  |
| Kenya | *Ae. aegypti* | Lab colony | *Aedes* partiti-like virus 1 | No | ISV | [13] |
| Uganda | *Ae. aegypti* | Lab colony | Formosus virus | No | ISV |  |
| Kenya | *Ae. aegypti* | Lab colony | Rabai virus | No | ISV |  |
| Côte d'Ivoire | *Ae. aegypti* | Field mosquitoes | Dezidougou virus | No | ISV | [14] |
| Kenya | *Ae. aegypti* | Field mosquitoes | Cell Fusing Agent Virus | No | ISV | [4] |
| Senegal | *Ae. aegypti* | Field mosquitoes | New *mesonivirus* (Dianke virus) | No | ISV | [15] |
| Côte d'Ivoire | *Ae. aegypti* | Field mosquitoes | *Alphamesonivirus* (Cavally virus) | No | ISV | [14] |
| Réunion | *Ae. albopictus* | Lab colony | *Cladosporidium* | No | Fungi | [2] |
| Réunion | *Ae. albopictus* | Lab colony | *Penicillium* | No | Fungi |  |
| Réunion | *Ae. albopictus* | Lab colony | *Parastagonospora* | No | Fungi |  |
| Réunion | *Ae. albopictus* | Lab colony | *Lasiodiplodia* | No | Fungi |  |
| Réunion | *Ae. albopictus* | Lab colony | *Malassezia* | No | Fungi |  |
| Réunion | *Ae. albopictus* | Lab colony | *Golovinomyces* | No | Fungi |  |
| Réunion | *Ae. albopictus* | Lab colony | *Aureobasidium* | No | Fungi |  |
| Réunion | *Ae. albopictus* | Lab colony | *Trichoderma* | No | Fungi |  |
| Réunion | *Ae. albopictus* | Lab colony | *Xylodon* | No | Fungi |  |
| Madagascar | *Ae. albopictus* | Field mosquitoes | *Cladosporidium* | No | Fungi | [16] |
| Madagascar | *Ae. albopictus* | Field mosquitoes | *Candida* | No | Fungi |  |
| Madagascar | *Ae. albopictus* | Field mosquitoes | *Hyphopichia* | No | Fungi |  |
| Madagascar | *Ae. albopictus* | Field mosquitoes | *Exobasidium* | No | Fungi |  |

References (Table S1)

1. Dickson LB, Ghozlane A, Volant S, Bouchier C, Ma L, Vega-Rúa A, et al. Diverse laboratory colonies of *Aedes aegypti* harbor the same adult midgut bacterial microbiome. Parasit Vectors. 2018;11:207. https://doi.org/10.1186/s13071-018-2780-1

2. Guégan M, Martin E, Valiente Moro C. Comparative Analysis of the bacterial and fungal communities in the gut and the crop of *Aedes albopictus* osquitoes: a preliminary study. Pathogens. Multidisciplinary Digital Publishing Institute; 2020;9:628. https://doi.org/10.3390/pathogens9080628

3. Scolari F, Sandionigi A, Carlassara M, Bruno A, Casiraghi M, Bonizzoni M. Exploring changes in the microbiota of *Aedes albopictus*: comparison among breeding site water, larvae, and adults. Front Microbiol. 2021;12:624170. https://doi.org/10.3389/fmicb.2021.624170

4. Ajamma YU, Onchuru TO, Ouso DO, Omondi D, Masiga DK, Villinger J. Vertical transmission of naturally occurring Bunyamwera and insect-specific flavivirus infections in mosquitoes from islands and mainland shores of Lakes Victoria and Baringo in Kenya. PLoS Negl Trop Dis. Public Library of Science; 2018;12:e0006949. https://doi.org/10.1371/journal.pntd.0006949

5. Bennouna A, Tantely ML, Raharinosy V, Andriamandimby SF, Bigot T, Chrétien D, et al. Comprehensive characterization of viral diversity of female mosquitoes in Madagascar. Viruses. Multidisciplinary Digital Publishing Institute; 2023;15:1852. https://doi.org/10.3390/v15091852

6. Chiuya T, Masiga DK, Falzon LC, Bastos ADS, Fèvre EM, Villinger J. A survey of mosquito-borne and insect-specific viruses in hospitals and livestock markets in western Kenya. PLOS ONE. Public Library of Science; 2021;16:e0252369. https://doi.org/10.1371/journal.pone.0252369

7. Guarido MM, Govender K, Riddin MA, Schrama M, Gorsich EE, Brooke BD, et al. Detection of insect-specific flaviviruses in mosquitoes (Diptera: Culicidae) in northeastern regions of South Africa. Viruses. 2021;13:2148. https://doi.org/10.3390/v13112148

8. Iwashita H, Higa Y, Futami K, Lutiali PA, Njenga SM, Nabeshima T, et al. Mosquito arbovirus survey in selected areas of Kenya: detection of insect-specific virus. Trop Med Health. 2018;46:19. https://doi.org/10.1186/s41182-018-0095-8

9. Oguzie JU, Nwangwu UC, Oluniyi PE, Olumade TJ, George UE, Kazeem A, et al. Metagenomic sequencing characterizes a wide diversity of viruses in field mosquito samples in Nigeria. Sci Rep. Nature Publishing Group; 2022;12:7616. https://doi.org/10.1038/s41598-022-11797-2

10. Proveti Olmo R, Todjro YMH, Aguiar ERGR, de Almeida JPP, Ferreira FV, Armache JN, et al. Mosquito vector competence for dengue is modulated by insect-specific viruses. Nat Microbiol. Nature Publishing Group; 2023;8:135–49. https://doi.org/10.1038/s41564-022-01289-4

11. Palatini U, Alfano N, Carballar-Lejarazu R, Chen X-G, Delatte H, Bonizzoni M. Virome and nrEVEome diversity of *Aedes albopictus* mosquitoes from La Reunion Island and China. Virol J. 2022;19:190. https://doi.org/10.1186/s12985-022-01918-8

12. Parry R, Asgari S. *Aedes* anphevirus: an insect-specific virus distributed worldwide in aedes aegypti mosquitoes that has complex interplays with wolbachia and dengue virus infection in cells. J Virol. American Society for Microbiology; 2018;92:10.1128/jvi.00224-18. https://doi.org/10.1128/jvi.00224-18

13. Parry R, James ME, Asgari S. Uncovering the worldwide diversity and evolution of the virome of the mosquitoes *Aedes aegypti* and *Aedes albopictus*. Microorganisms. Multidisciplinary Digital Publishing Institute; 2021;9:1653. https://doi.org/10.3390/microorganisms9081653

14. Vasilakis N, Guzman H, Firth C, Forrester NL, Widen SG, Wood TG, et al. Mesoniviruses are mosquito-specific viruses with extensive geographic distribution and host range. Virol J. 2014;11:97. https://doi.org/10.1186/1743-422X-11-97

15. Diagne MM, Gaye A, Ndione MHD, Faye M, Fall G, Dieng I, et al. Dianke virus: A new mesonivirus species isolated from mosquitoes in eastern Senegal. Virus Res. 2020;275:197802. https://doi.org/10.1016/j.virusres.2019.197802

16. Luis P, Vallon L, Tran F-H, Hugoni M, Tran-Van V, Mavingui P, et al. *Aedes albopictus* mosquitoes host a locally structured mycobiota with evidence of reduced fungal diversity in invasive populations. Fungal Ecol. 2019;39:257–66. https://doi.org/10.1016/j.funeco.2019.02.004

Table S2 Challenges of using microbiota to fight *Ae. aegypti*

| Gaps of knowledge | Comments |
| --- | --- |
| Mechanisms of microbial acquisition and maintenance | Understanding how certain microbial species are acquired and maintained in host mosquitoes, particularly those with high vector control potential. |
| Microbiota variability factors | Limited knowledge about the influence of the environment, diet, and other factors on the diversity of the microbiota in *Ae. aegypti* limits the generalization of the effects of microbiota on pathogen transmission. |
| Mechanisms of microbiota-pathogen interaction | A lack of comprehension regarding the interaction between bacteria, viruses, or fungi and arboviruses hinders our ability to influence their transmission. This thereby hinders the targeted exploitation of these interactions. |
| Environmental and evolutionary impacts | Need to assess ecological risks, such as horizontal gene transfer and the potential evolution of resistance in mosquito populations or microbiota, which could undermine control strategies. |
| Microbiota dynamics in natural populations | Limited data on how microbiota is maintained over generations and how environmental changes affect their stability and composition in natural mosquito populations. |
| Integration with other control strategies | Lack of comprehensive studies on how microbiota-based interventions synergize or interfere with existing methods like insecticides or habitat elimination, affecting overall effectiveness. |
| Large-scale implementation challenges | Requirements for robust infrastructure, monitoring systems, and societal acceptance to effectively deploy microbiota-based strategies at scale remain underdeveloped. |
