# Supplementary material for: Unlocking new frontiers in vector control strategies using Aedes aegypti microbiota
Source: Parasit Vectors. 2026 May 5;19:260. doi: 10.1186/s13071-026-07304-5 (PMC13289545; doi:10.1186/s13071-026-07304-5)
Supplement: Supplementary file 2 — Additional file 2. [file 13071_2026_7304_MOESM2_ESM.docx]

Table S3. Key microbiota target identified in *Aedes aegypti* across the world

Dataset was adapted from Mantilla-Granados et al. (2024) [58] supplementary materials and update by us. AF: Africa; AS: Asia; EU: Europa; NA: North America; OC: Oceania; SA: South America; ISV: Insect-specific virus

| Continent | Target | Microbial type | References |
| --- | --- | --- | --- |
| AF | *Pseudoalteromonas* | Bacterial | Dickson et al., 2018 |
| AF | *Pseudomonas* | Bacterial | Dickson et al., 2018 |
| AF | *Luteibacter* | Bacterial | Dickson et al., 2018 |
| AF | *Rhodanobacter* | Bacterial | Dickson et al., 2018 |
| AF | *Enterobacter* | Bacterial | Dickson et al., 2018 |
| AF | *Pseudomonas* | Bacterial | Dickson et al., 2018 |
| AF | *Pseudoalteromonas* | Bacterial | Dickson et al., 2018 |
| AF | *Luteibacter* | Bacterial | Dickson et al., 2018 |
| AF | *Rhodanobacter* | Bacterial | Dickson et al., 2018 |
| AF | *Marinomonas* | Bacterial | Dickson et al., 2018 |
| AF | Cell fusing agent virus | ISV | Ajamma et al., 2018 |
| AF | Guadeloupe Culex rhabdovirus | ISV | Bennouna et al., 2023 |
| AF | Grenada mosquito rhabdovirus 1 | ISV | Bennouna et al., 2023 |
| AF | Xiang Yun picorna-like virus 4 | ISV | Bennouna et al., 2023 |
| AF | Aedes flavivirus | ISV | Chiuya et al., 2021 |
| AF | Cell fusing agent virus | ISV | Chiuya et al., 2021 |
| AF | Cell fusing agent virus | ISV | Guarido et al., 2021 |
| AF | Cell fusing agent virus | ISV | Iwashita et al., 2018 |
| AF | Aedes aegypti toti virus | ISV | Oguzie et al., 2022 |
| AF | Cell fusing agent virus | ISV | Oguzie et al., 2022 |
| AF | Chaq-Like virus | ISV | Oguzie et al., 2022 |
| AF | Fako virus | ISV | Oguzie et al., 2022 |
| AF | Phasi Charoen-like phasivirus | ISV | Oguzie et al., 2022 |
| AF | Tesano Aedes Virus | ISV | Oguzie et al., 2022 |
| AF | Aslam narnavirus | ISV | Olmo et al., 2023 |
| AF | Humaita-Tubiacanga virus | ISV | Olmo et al., 2023 |
| AF | Phasi Charoen-like virus | ISV | Olmo et al., 2023 |
| AF | Aedes anphevirus | ISV | Parry and Asgari , 2018 |
| AF | Aedes anphevirus | ISV | Parry and Asgari , 2018 |
| AF | Aedes partiti-like virus 1 | ISV | Parry et al., 2021 |
| AF | Formosus virus | ISV | Parry et al., 2021 |
| AF | Rabai virus | ISV | Parry et al., 2021 |
| AF | Dezidougou virus | ISV | Vasilakis et al., 2014 |
| AF | Cell Fusing Agent Virus | ISV | Ajamma et al., 2018 |
| AF | New mesonivirus (Dianke virus) | ISV | Diagne et al., 2020 |
| AF | Alphamesonivirus (Cavally virus) | ISV | Vasilakis et al., 2014 |
| AS | *Bacillus* | Bacterial | Al-Ghamdi et al., 2023 |
| AS | *Lysinibacillus* | Bacterial | Al-Ghamdi et al., 2023 |
| AS | *Bacillus* | Bacterial | Balaji et a., 2021 |
| AS | *Chryseobacterium* | Bacterial | Balaji et a., 2021 |
| AS | *Elizabethkingia* | Bacterial | Balaji et a., 2021 |
| AS | *Pantoea* | Bacterial | Balaji et a., 2021 |
| AS | *Microbacterium* | Bacterial | Balaji et a., 2021 |
| AS | *Serratia* | Bacterial | Balaji et a., 2021 |
| AS | *Enterobacter* | Bacterial | Balaji et a., 2021 |
| AS | *Enterococcus* | Bacterial | Balaji et a., 2021 |
| AS | *Undibacterium* | Bacterial | Balaji et a., 2021 |
| AS | *Wolbachia* | Bacterial | Balaji et a., 2021 |
| AS | *Wolbachia* | Bacterial | Balaji et al., 2019 |
| AS | *Wolbachia* | Bacterial | Carvajal et al., 2019 |
| AS | *Wolbachia* | Bacterial | Chao et al., 2023 |
| AS | *Pseudomonas* | Bacterial | Dickson et al., 2018 |
| AS | *Pseudoalteromonas* | Bacterial | Dickson et al., 2018 |
| AS | *Luteibacter* | Bacterial | Dickson et al., 2018 |
| AS | *Rhodanobacter* | Bacterial | Dickson et al., 2018 |
| AS | *Marinomonas* | Bacterial | Dickson et al., 2018 |
| AS | *Methylobacterium* | Bacterial | Lin et al., 2021 |
| AS | *Enterobacter* | Bacterial | Lin et al., 2021 |
| AS | *Ralstonia* | Bacterial | Lin et al., 2021 |
| AS | *Leptothrix* | Bacterial | Lin et al., 2021 |
| AS | *Asaia* | Bacterial | Lin et al., 2021 |
| AS | *Elizabethkingia* | Bacterial | Martinez et al., 2023 |
| AS | *Phyllobacterium* | Bacterial | Martinez et al., 2023 |
| AS | *Asaia* | Bacterial | Martinez et al., 2023 |
| AS | *Bacillus* | Bacterial | Ranasinghe et al., 2021 |
| AS | *Staphylococcus* | Bacterial | Ranasinghe et al., 2021 |
| AS | *Enterobacter* | Bacterial | Ranasinghe et al., 2021 |
| AS | *Pantoea* | Bacterial | Ranasinghe et al., 2021 |
| AS | *Acinetobacter* | Bacterial | Ranasinghe et al., 2021 |
| AS | *Aeromonas* | Bacterial | Rodpai et al., 2023 |
| AS | *Bosea* | Bacterial | Rodpai et al., 2023 |
| AS | *clostridioides* | Bacterial | Rodpai et al., 2023 |
| AS | *Klebsiella* | Bacterial | Rodpai et al., 2023 |
| AS | *Escherichia* | Bacterial | Rodpai et al., 2023 |
| AS | *Acinetobacter* | Bacterial | Sarma et al., 2022 |
| AS | *Caulobacter* | Bacterial | Sarma et al., 2022 |
| AS | *Bacillus* | Bacterial | Sarma et al., 2022 |
| AS | *Brevandimonas* | Bacterial | Sarma et al., 2022 |
| AS | *Pseudomonas* | Bacterial | Sarma et al., 2022 |
| AS | *Serratia* | Bacterial | Sarma et al., 2022 |
| AS | *Wolbachia* | Bacterial | Somia et al., 2023 |
| AS | *Wolbachia* | Bacterial | Thongsripong et al., 2018 |
| AS | *Wolbachia* | Bacterial | Thongsripong et al., 2018 |
| AS | *Erwinia* | Bacterial | Thongsripong et al., 2018 |
| AS | *Pseudomonas* | Bacterial | Thongsripong et al., 2018 |
| AS | *Stenotrophomonas* | Bacterial | Thongsripong et al., 2018 |
| AS | *Serratia* | Bacterial | Thongsripong et al., 2018 |
| AS | *Enterobacter* | Bacterial | Thongsripong et al., 2018 |
| AS | *Comamonas* | Bacterial | Thongsripong et al., 2018 |
| AS | *Wolbachia* | Bacterial | Vinayagam et al., 2023 |
| AS | *Enterobacter* | Bacterial | Yadav et al., 2015 |
| AS | *Pseudomonas* | Bacterial | Yadav et al., 2015 |
| AS | *Lysinibacillus* | Bacterial | Yadav et al., 2015 |
| AS | *Staphylococcus* | Bacterial | Yadav et al., 2015 |
| AS | *Stenotrophomonas* | Bacterial | Yadav et al., 2015 |
| AS | *Pantoea* | Bacterial | Yadav et al., 2015 |
| AS | *Klebsiella* | Bacterial | Zakrzewski et al., 2018 |
| AS | *Enterobacter* | Bacterial | Zakrzewski et al., 2018 |
| AS | *Wolbachia* | Bacterial | Zhang et al., 2022 |
| AS | *Fusarium* | Fungi | Zakrzewski et al., 2018 |
| AS | Cell fusing agent virus | ISV | Baidaliuk et al., 2020 |
| AS | Phasi Charoen-like virus | ISV | Chandler et al., 2015 |
| AS | Aedes flavivirus | ISV | Fang et al ., 2021 |
| AS | Cell fusing agent virus | ISV | Fang et al ., 2021 |
| AS | Aedes flavivirus | ISV | Fang et al., 2021 |
| AS | Circovirus | ISV | Hameed et al., 2021 |
| AS | Culex Bunya-like virus | ISV | Hameed et al., 2021 |
| AS | Hubei arthropod virus 1 | ISV | Hameed et al., 2021 |
| AS | Hubei mosquito virus 2 | ISV | Hameed et al., 2021 |
| AS | Wenzhou sobemo-like virus 3 | ISV | Hameed et al., 2021 |
| AS | Yongsan tombus-like viru 1 | ISV | Hameed et al., 2021 |
| AS | Phasi Charoen-like phasivirus | ISV | Munivenkatappa et al., 2021 |
| AS | Aedes anphevirus | ISV | Olmo et al., 2023 |
| AS | Guadeloupe mosquito virus | ISV | Olmo et al., 2023 |
| AS | Humaita-Tubiacanga virus | ISV | Olmo et al., 2023 |
| AS | Nyamuk partiti-like virus | ISV | Olmo et al., 2023 |
| AS | Phasi Charoen-like virus | ISV | Olmo et al., 2023 |
| AS | Aedes anphevirus | ISV | Parry and Asgari , 2018 |
| AS | Aedes anphevirus | ISV | Parry and Asgari , 2018 |
| AS | Aedes anphevirus | ISV | Parry and Asgari , 2018 |
| AS | Aedes binegev-like virus 1 | ISV | Parry et al., 2021 |
| AS | Aedes binegev-like virus 1 | ISV | Parry et al., 2021 |
| AS | Aedes partiti-like virus 1 | ISV | Parry et al., 2021 |
| AS | Aedes partiti-like virus 1 | ISV | Parry et al., 2021 |
| AS | Aedes partiti-like virus 1 | ISV | Parry et al., 2021 |
| AS | Aedes partiti-like virus 1 | ISV | Parry et al., 2021 |
| AS | Aedes partiti-like virus 1 | ISV | Parry et al., 2021 |
| AS | Banna virus | ISV | Supriyono et al., 2020 |
| AS | Bogor virus | ISV | Supriyono et al., 2020 |
| AS | Cell fusing agent virus | ISV | Supriyono et al., 2020 |
| AS | Mosquito flavivirus | ISV | Yezli et al ., 2021 |
| AS | Aedes aegypti densovirus 2 | ISV | Zakrzewski et al., 2018 |
| AS | Aedes aegypti toti-like virus | ISV | Zakrzewski et al., 2018 |
| AS | Cell fusing agent virus | ISV | Zakrzewski et al., 2018 |
| AS | Flavivirus | ISV | Zakrzewski et al., 2018 |
| AS | Humaita-Tubiacanga virus | ISV | Zakrzewski et al., 2018 |
| AS | Orthomyxovirus | ISV | Zakrzewski et al., 2018 |
| AS | Phasi Charoen-like phasivirus | ISV | Zakrzewski et al., 2018 |
| AS | Phlebovirus | ISV | Zakrzewski et al., 2018 |
| AS | Whidbey virus | ISV | Zakrzewski et al., 2018 |
| AS | Phasi Charoen-like virus | ISV | Zhang et al., 2018 |
| EU | Aedes binegev-like virus 1 | ISV | Parry et al., 2021 |
| NA | *Wolbachia* | Bacterial | Kulkarni et al., 2018 |
| NA | *Elizabethkingia* | Bacterial | Bennett et al., 2019 |
| NA | *Morganella* | Bacterial | Bennett et al., 2019 |
| NA | *Serratia* | Bacterial | Bennett et al., 2019 |
| NA | *Acinetobacter* | Bacterial | Bennett et al., 2019 |
| NA | *Pseudomonas* | Bacterial | Bennett et al., 2019 |
| NA | *Stenotrophomonas* | Bacterial | Bennett et al., 2019 |
| NA | *Pseudomonas* | Bacterial | Dickson et al., 2018 |
| NA | *Pseudoalteromonas* | Bacterial | Dickson et al., 2018 |
| NA | *Luteibacter* | Bacterial | Dickson et al., 2018 |
| NA | *Rhodanobacter* | Bacterial | Dickson et al., 2018 |
| NA | *Marinomonas* | Bacterial | Dickson et al., 2018 |
| NA | *Pseudomonas* | Bacterial | Hegde et al., 2018 |
| NA | *Zymobacter* | Bacterial | Hegde et al., 2018 |
| NA | *Tatumella* | Bacterial | Hegde et al., 2018 |
| NA | *Serratia* | Bacterial | Hegde et al., 2018 |
| NA | *Enterobacter* | Bacterial | Hegde et al., 2018 |
| NA | *Asaia* | Bacterial | Hegde et al., 2018 |
| NA | *Escherichia* | Bacterial | Ramos-Nino et al., 2020 |
| NA | *Actinomyces* | Bacterial | Ramos-Nino et al., 2020 |
| NA | *Serratia* | Bacterial | Ramos-Nino et al., 2020 |
| NA | *Zymobacter* | Bacterial | Ramos-Nino et al., 2020 |
| NA | *Pseudomonas* | Bacterial | Ramos-Nino et al., 2020 |
| NA | *Spironema* | Bacterial | Ramos-Nino et al., 2020 |
| NA | *Stenotrophomonas* | Bacterial | Ramos-Nino et al., 2020 |
| NA | *Acinetobacter* | Bacterial | Ramos-Nino et al., 2020 |
| NA | *Carninomonas* | Bacterial | Ramos-Nino et al., 2020 |
| NA | *Asaia* | Bacterial | Ramos-Nino et al., 2020 |
| NA | *Talaromyces* | Fungi | Angleró-Rodríguez et al., 2017 |
| NA | *Aspergillus* | Fungi | Ramos-Nino et al., 2029 |
| NA | *Edhazardia* | Fungi | Ramos-Nino et al., 2029 |
| NA | *Nosema* | Fungi | Ramos-Nino et al., 2029 |
| NA | *Malassezia* | Fungi | Ramos-Nino et al., 2029 |
| NA | *Aedes aegypti* toti-like viru*s* | ISV | Batson et al., 2021 |
| NA | *Aedes* anphevirus | ISV | Batson et al., 2021 |
| NA | Barstukas virus | ISV | Batson et al., 2021 |
| NA | Guadeloupe mosquito quaranja-like virus 1 | ISV | Batson et al., 2021 |
| NA | Guadeloupe mosquito virus | ISV | Batson et al., 2021 |
| NA | Usinis virus | ISV | Batson et al., 2021 |
| NA | Wuhan insect virus 33 | ISV | Batson et al., 2021 |
| NA | orthomyxo-like viruse | ISV | Coatsworth et al., 2022 |
| NA | partiti-like virus | ISV | Coatsworth et al., 2022 |
| NA | Toti-like virus | ISV | Coatsworth et al., 2022 |
| NA | Cell fusing agent virus | ISV | Fang et al., 2021 |
| NA | Cell fusing agent virus | ISV | Jeffries et al., 2020 |
| NA | Cell fusing agent virus | ISV | Martin et al., 2019 |
| NA | Cell fusing agent virus | ISV | Martin et al., 2020 |
| NA | Aedes anphevirus | ISV | Parry and Asgari , 2018 |
| NA | Aedes anphevirus | ISV | Parry and Asgari , 2018 |
| NA | Aedes anphevirus | ISV | Parry and Asgari , 2018 |
| NA | Aedes binegev-like virus 1 | ISV | Parry et al., 2021 |
| NA | Aedes binegev-like virus 1 | ISV | Parry et al., 2021 |
| NA | Aedes binegev-like virus 1 | ISV | Parry et al., 2021 |
| NA | Aedes partiti-like virus 1 | ISV | Parry et al., 2021 |
| NA | Aedes partiti-like virus 1 | ISV | Parry et al., 2021 |
| NA | Aedes partiti-like virus 1 | ISV | Parry et al., 2021 |
| NA | Aedes partiti-like virus 1 | ISV | Parry et al., 2021 |
| NA | Aedes partiti-like virus 1 | ISV | Parry et al., 2021 |
| NA | Anphevirus | ISV | Ramos-Nino et al., 2029 |
| NA | Phasivirus | ISV | Ramos-Nino et al., 2029 |
| NA | Aedes anphevirus | ISV | Shi et al., 2019 |
| NA | Anopheles totivirus | ISV | Shi et al., 2019 |
| NA | Chuvirus | ISV | Shi et al., 2019 |
| NA | Hubei toti-like virus | ISV | Shi et al., 2019 |
| NA | Humaita-Tubiacanga virus | ISV | Shi et al., 2019 |
| NA | Kaiowa virus | ISV | Shi et al., 2019 |
| NA | Menghai flavivirus | ISV | Shi et al., 2019 |
| NA | Phasi Charoen-like phasivirus | ISV | Shi et al., 2019 |
| NA | Trichoplusia ni TED virus | ISV | Shi et al., 2019 |
| NA | Whenzhou sobermo-like virus | ISV | Shi et al., 2019 |
| NA | Alphamesonivirus 1 | ISV | Thannesberger et al., 2020 |
| NA | Anopheles gambiae densovirus | ISV | Thannesberger et al., 2020 |
| NA | Anopheles totivirus | ISV | Thannesberger et al., 2020 |
| NA | Humaita-Tubiacanga virus | ISV | Thannesberger et al., 2020 |
| NA | Phasi Charoen-like phasivirus | ISV | Thannesberger et al., 2020 |
| NA | Wenzhou soberno-like virus 4 | ISV | Thannesberger et al., 2020 |
| OC | *Pseudomonas* | Bacterial | Dickson et al., 2018 |
| OC | *Pseudoalteromonas* | Bacterial | Dickson et al., 2018 |
| OC | *Luteibacter* | Bacterial | Dickson et al., 2018 |
| OC | *Rhodanobacter* | Bacterial | Dickson et al., 2018 |
| OC | *Enterobacter* | Bacterial | Dickson et al., 2018 |
| OC | *Aeromonas* | Bacterial | Zakrzewski et al., 2018 |
| OC | *Bacillus* | Bacterial | Zakrzewski et al., 2018 |
| OC | *Brevibacterium* | Bacterial | Zakrzewski et al., 2018 |
| OC | *Proteobacteria* | Bacterial | Zakrzewski et al., 2018 |
| OC | *Pantoea* | Bacterial | Zakrzewski et al., 2018 |
| OC | *Edhazardia* | Fungi | Zakrzewski et al., 2018 |
| OC | *Penicilium* | Fungi | Zakrzewski et al., 2018 |
| OC | Aedes anphevirus | ISV | Parry and Asgari , 2018 |
| OC | Aedes orbi-like virus | ISV | Parry et al., 2021 |
| OC | Aedes orbi-like virus | ISV | Parry et al., 2021 |
| OC | Aedes partiti-like virus 1 | ISV | Parry et al., 2021 |
| OC | Cell fusing agent virus | ISV | Zakrzewski et al., 2018 |
| OC | Humaita-Tubiacanga virus | ISV | Zakrzewski et al., 2018 |
| OC | Phasi Charoen-like phasivirus | ISV | Zakrzewski et al., 2018 |
| SA | *Bacteroides* | Bacterial | Arévalo-Cortés et al., 2020 |
| SA | *Clostridium* | Bacterial | Arévalo-Cortés et al., 2020 |
| SA | *Prevotella* | Bacterial | Arévalo-Cortés et al., 2020 |
| SA | *Akkermansia* | Bacterial | Arévalo-Cortés et al., 2020 |
| SA | *Parabacteroides* | Bacterial | Arévalo-Cortés et al., 2020 |
| SA | *Ruminococcus* | Bacterial | Arévalo-Cortés et al., 2020 |
| SA | *Roseburia* | Bacterial | Arévalo-Cortés et al., 2020 |
| SA | *Bifidobacterium* | Bacterial | Arévalo-Cortés et al., 2020 |
| SA | *Faecalibacterium* | Bacterial | Arévalo-Cortés et al., 2020 |
| SA | *Megasphaera* | Bacterial | Arévalo-Cortés et al., 2020 |
| SA | *Pseudoalteromonas* | Bacterial | Dickson et al., 2018 |
| SA | *Pseudomonas* | Bacterial | Dickson et al., 2018 |
| SA | *Luteibacter* | Bacterial | Dickson et al., 2018 |
| SA | *Rhodanobacter* | Bacterial | Dickson et al., 2018 |
| SA | *Intestinibacter* | Bacterial | Dickson et al., 2018 |
| SA | *Pseudomonas* | Bacterial | Molina-Henao et al., 2020 |
| SA | *Serratia* | Bacterial | Molina-Henao et al., 2020 |
| SA | *Escherichia* | Bacterial | Molina-Henao et al., 2020 |
| SA | *Stenotrophomonas* | Bacterial | Molina-Henao et al., 2020 |
| SA | *Candida* | Bacterial | Molina-Henao et al., 2020 |
| SA | *Bacillus* | Bacterial | Molina-Henao et al., 2020 |
| SA | *Aedes aegypti* toti-like virus | ISV | Calle-Tobón et al., 2022 |
| SA | *Aedes aegypti* virga-like virus | ISV | Calle-Tobón et al., 2022 |
| SA | *Aedes* anphevirus | ISV | Calle-Tobón et al., 2022 |
| SA | Australian *Anopheles* totivirus | ISV | Calle-Tobón et al., 2022 |
| SA | Cell fusing agent virus | ISV | Calle-Tobón et al., 2022 |
| SA | Guadeloupe mosquito guaranja-like virus | ISV | Calle-Tobón et al., 2022 |
| SA | Guadeloupe mosquito virus | ISV | Calle-Tobón et al., 2022 |
| SA | Humaita-Tubiacanga virus | ISV | Calle-Tobón et al., 2022 |
| SA | Kwale mosquito virus | ISV | Calle-Tobón et al., 2022 |
| SA | Phasi Charoen-like phasivirus | ISV | Calle-Tobón et al., 2022 |
| SA | Phasi Charoen-like virus | ISV | Cunha et al., 2020 |
| SA | Alphamesonivirus 1 | ISV | da Silva Ferreira et al., 2020 |
| SA | Culex circovirus-like virus | ISV | da Silva Ferreira et al., 2020 |
| SA | Culex flavivirus | ISV | da Silva Ferreira et al., 2020 |
| SA | Culex hubei-like virus | ISV | da Silva Ferreira et al., 2020 |
| SA | Culex Iflavi-like virus 4 | ISV | da Silva Ferreira et al., 2020 |
| SA | Hubei mosquito virus 4 | ISV | da Silva Ferreira et al., 2020 |
| SA | Nam Dinh virus | ISV | da Silva Ferreira et al., 2020 |
| SA | Phasi Charoen-like virus | ISV | da Silva Ferreira et al., 2020 |
| SA | Wuhan mosquito virus 4 | ISV | da Silva Ferreira et al., 2020 |
| SA | Wutai mosquito phasivirus | ISV | da Silva Ferreira et al., 2020 |
| SA | Aedes aegypti To virus 1 | ISV | Duarte et al., 2023 |
| SA | Aedes aegypti To virus 2 | ISV | Duarte et al., 2023 |
| SA | Phasi Charoen-like phasivirus | ISV | Duarte et al., 2023 |
| SA | Cell fusing agent virus | ISV | Fernandes et al., 2018 |
| SA | Cell fusing agent virus | ISV | Olmo et al., 2023 |
| SA | Guadeloupe mosquito virus | ISV | Olmo et al., 2023 |
| SA | Humaita-Tubiacanga virus | ISV | Olmo et al., 2023 |
| SA | Orbis virgavirus | ISV | Olmo et al., 2023 |
| SA | Phasi Charoen-like phasivirus | ISV | Olmo et al., 2023 |
| SA | Aedes anphevirus | ISV | Parry and Asgari , 2018 |
| SA | Aedes anphevirus | ISV | Parry and Asgari , 2018 |
| SA | Aedes binegev-like virus 1 | ISV | Parry et al., 2021 |
| SA | Aedes Iflavi-like virus 1 | ISV | Ribeiro et al., 2020 |
| SA | Aedes Iflavi-like virus 2 | ISV | Ribeiro et al., 2020 |
| SA | Aedes permutotetra-like virus 1 | ISV | Ribeiro et al., 2020 |
| SA | Aedes permutotetra-like virus 2 strain AP59 | ISV | Ribeiro et al., 2020 |
| SA | Aedes permutotetra-like virus 2 strain AP60 | ISV | Ribeiro et al., 2020 |
| SA | Aedes Sobemo-like virus strain AP60-1 | ISV | Ribeiro et al., 2020 |
| SA | Aedes Sobemo-like virus strain AP60-2 | ISV | Ribeiro et al., 2020 |
| SA | *Acetobacter* | Bacterial | Gomez et al.,2025 |
| SA | *Bacillus* | Bacterial | Gomez et al.,2025 |
